# The prevalence of using beta-blockers and its relationship with social anxiety among health profession students at Umm Al-Qura University

**DOI:** 10.1371/journal.pone.0307163

**Published:** 2024-08-13

**Authors:** Baraa Sami Quronfulah, Ruyuf Saleh Alzahrani, Ebtesam Tariq Kattan, Hala Mamun Tamim, Taif Hazzaa Alharbi, Mariyyah Mohammed Alghamdi, Amal Mohammad Badawoud

**Affiliations:** 1 Department of Health Promotion and Health Education, College of Public Health and Health Informatics, Umm Al-Qura University, Makkah, Saudi Arabia; 2 Department of Pharmacy Practice, College of Pharmacy, Princess Nourah Bint Abdulrahman University, Riyadh, Saudi Arabia; Ahmed Physiotherapy and Research Center, BANGLADESH

## Abstract

**Background:**

Social Anxiety Disorder (SAD) is an anxiety disorder characterized by excessive fear of scrutiny in social situations. Health students are more susceptible to SAD due to academic demands. They may resort to self-medication, particularly beta-blockers (BBs) for managing physical symptoms of SAD. The study aims to investigate the prevalence of beta-blocker use and its relationship with social anxiety disorder among health students at Umm Al-Qura University.

**Methods:**

In this cross-sectional study, 461 undergraduate health students participated in a questionnaire with 30 questions divided into three sections: The Social Phobia Inventory (SPIN), BBs usage behavior questionnaire, and demographic characteristics.

**Results:**

The study found 56.2% had SAD. A total of 7.8% of the sample reported using BBs, and no significant correlation was found between the usage of BBs and the SAD score (P = 0.085).

**Conclusion:**

The study revealed significant relationships between the presence of SAD with gender, history of mental conditions, and correlation between the use of BBs with history of mental conditions. Although BBs usage is low among health students, the prevalence of SAD is alarming. The results could raise awareness about the need for early detection of SAD among health students.

## Introduction

Social Anxiety Disorder (SAD) is an individual’s fear of social situations where they may be judged negatively [1]. This fear can have a detrimental impact on the quality of life and daily activities of people with SAD, leading to social isolation and limited educational and occupational opportunities [2]. Healthcare students are at a higher risk of developing SAD due to the elevated stress levels associated with their rigorous academic requirements, clinical rotations, and interpersonal training. To cope with this stress, some students resort to using beta-blockers (BB) (propranolol) beyond its intended medical use to manage physical symptoms and alleviate stress in performance situations. However, the misuse of BB without a prescription is a concern, as it may have unintended consequences. By raising awareness among health students about the potential association between BB use and SAD, this study holds significance for the target population.

SAD is the third most common mental disorder worldwide after depression and alcohol abuse [3]. In the United States, SAD is the second most diagnosed anxiety disorder, affecting 15 million adults [4]. These findings are relevant to local research conducted in Saudi Arabia, which indicates a high prevalence of SAD with a lifetime prevalence rate of 5.6% among total cases [5].

Health profession students, who experience heightened academic pressure and societal problems, are at a significantly increased risk of developing mental health issues [6]. Among these students, 8.21% and 4.21% experience severe and very severe SAD, respectively [7]. A study found that 23% of health students had used BBs, such as propranolol, atenolol, pindolol, nadolol, and oxprenolol, to alleviate anxiety symptoms during their academic years [8, 9]. Notably, propranolol stands out from the other four BBs as it can also reduce palpitations [10]. Its use is particularly common among medical students due to its potential to improve academic performance [11]. The reliance on medications to control anxiety symptoms could lead to dependence, limited effectiveness over time, and potential side effects. It is imperative to incorporate therapy, lifestyle changes, and other non-pharmacological interventions to manage anxiety effectively.

The hypothesis is that health students at Umm Al-Qura University (UQU) who have been diagnosed with social anxiety disorder are more likely to use BBs than those without such a diagnosis, indicating a potential relationship between BB use and SAD prevalence among this population. The primary objective of our study was to investigate the prevalence of BB use and its relationship with SAD among health students at UAU.

## Materials and methods

### Study design and population

The authors of this study used a cross-sectional study at UQU, Makkah, Saudi Arabia, the participant pool comprised university undergraduate students from health colleges including all health students in UQU, regardless of gender, college, and level. However, all staff members, including academic, medical, and administrative staff, were excluded. All participants provided informed consent to enter the study protocol.

### Sample size

The required sample size was calculated using Cochran’s Sample Size Formula for Proportion [12]. The minimum required sample size calculation of a confidence interval of 95%, a margin of error of 5%, and a population proportion of 50% is 385 students. This number 385 was amplified by 15% for the non-response rate yielding that a sample size of 443 students was needed for this study.

### Procedure

Data were collected from February 26, 2023 to May 5, 2023. The present study used questionnaire distributed to the potential participants. The questionnaire was electronically uploaded to the UQU website and shared on social networking sites with a statement outlining the study’s objectives, identifying the target audience, and ensuring the confidentiality of responses. In addition, the questionnaire was disseminated through leaders in specific health student groups, UQU’s student clubs for health students, and directly distributed to friends from various health specialties. To increase the participation rate, an animation video was made.

### Questionnaire development

The study utilized a voluntarily self-administered questionnaire accessible to participants through their university’s student account. The questionnaire consisted of 30 questions divided into three sections. The first section includes an Arabic version of The Social Phobia Inventory (SPIN) which contains seventeen items for screening and measuring the severity of SAD (Social Phobia—SP) [13]. The scale’s internal consistency ranged between 0.74 and 0.89 for all 17 items, and the 3-week test-retest reliability was 0.89 in a sample of 160 students in Saudi Arabia [13]. In the current study, Cronbach’s alpha was excellent at 0.94. Each item was calculated on a 5-point Likert scale, ranging from 0 (not at all) to 4 (extremely). Respondents indicated how much each item bothered them during the past week. The second section of the study comprises six multiple-choice questions about BB usage patterns, which consist of dosage, prescription, frequency, and reasons for usage. These questions were extracted from a previous study conducted by Alkhatabi in 2020 and have been meticulously refined for accuracy and clarity of understanding [14]. The third section was seven demographic characteristics questions which consisted of gender, college, academic year, chronic diseases, psychological and mental disorders, and Grade Point Average (GPA).

### Statistical analysis

All statistical analyses were conducted using Statistical Package for Social Sciences (SPSS) (Version 25; IBM Corp, Armonk, NY). The data was transferred from an Excel sheet to the SPSS program and then processed and analyzed into detailed tables. All calculations were performed twice and on different days to eliminate possible calculation errors. Continuous data were reported as means and standard deviations, while categorical data were reported as observation counts and proportions. For bivariate analysis, Chi-square test or Fisher’s Exact test was performed. Statistical significance was defined as P ≤ 0.05 (two-tailed).

### Ethics

Approval for the study was granted by The Biomedical Research Ethics Committee at UQU, Makkah, Saudi Arabia in February 2023, with the approval number HAPO-02-K-012-2023-02-1478. There was a low risk of harm with the participants completing the survey beyond everyday living, which is giving up their time to complete the survey. All participants were informed about the study’s aims and objectives before participating. As all data was unidentifiable, participants were informed that their willingness to return the completed questionnaire indicated their consent to participate in the study.

Maximum efforts were used to protect participants’ anonymity and confidentiality. Personal information such as names or contact information was not gathered, ensuring the privacy of all participants. All unidentifiable data were stored in a password-locked computer and only available to the research team.

## Results

### Demographic characteristics of participants

Four hundred sixty-one students participated in the study, with 46.2% in their first and second years, 42.7% in their third and fourth years, and 11.1% in their fifth, sixth, and seventh years. Almost two-thirds of students (57.7%) were from Public Health and Health Informatics (PHHI) College, while the remaining 42.3% were from Applied Medical Sciences, Nursing, Pharmacy, Medicine, and Dentistry. Regarding their grades, 66.8% of the students had a GPA ranging from 4.00 to 3.50, while 33.2% had a GPA below 3.50 (See Table 1).

**Table 1:** Distribution of demographic characteristics of the participants (n = 461).

Demographical Characteristics	Frequency	Percent (%)
**Gender**		
Female	403	87.4
Male	58	12.6
**Colleges**		
Public Health and Health Informatics	266	57.7
Other Health Colleges	195	42.3
**Academic Year**		
1^st^ and 2^nd^ year	213	46.2
3^rd^ and 4^th^ year	197	42.7
5^th^, 6^th^, and 7^th^ year	51	11.1
**GPA**		
Excellent (3.50–4.00)	308	66.8
Good To Very good (1.75–3.49)	153	33.2

### Social Phobia Inventory scores

A total of 259 students (56.2%) exhibited some degree of Social Phobia. More specifically, 181 students (39.3%) reported mild to moderate levels of Social Phobia, whereas 78 students (16.9%) indicated experiencing severe to very severe levels of Social Phobia (See Table 2).

**Table 2:** Distribution of Social Phobia Inventory scores and beta-blocker usage (n = 461).

	Frequency	Percent (%)
**Social Phobia Inventory Scores**		
No SP	202	43.8
Mild to Moderate SP	181	39.3
Severe to Very Severe SP	78	16.9
**Beta-Blocker Use**		
Yes	36	7.8
No	425	92.2

### Beta-blocker usage frequency, daily dosage, and prescription source

**Fig 1:**
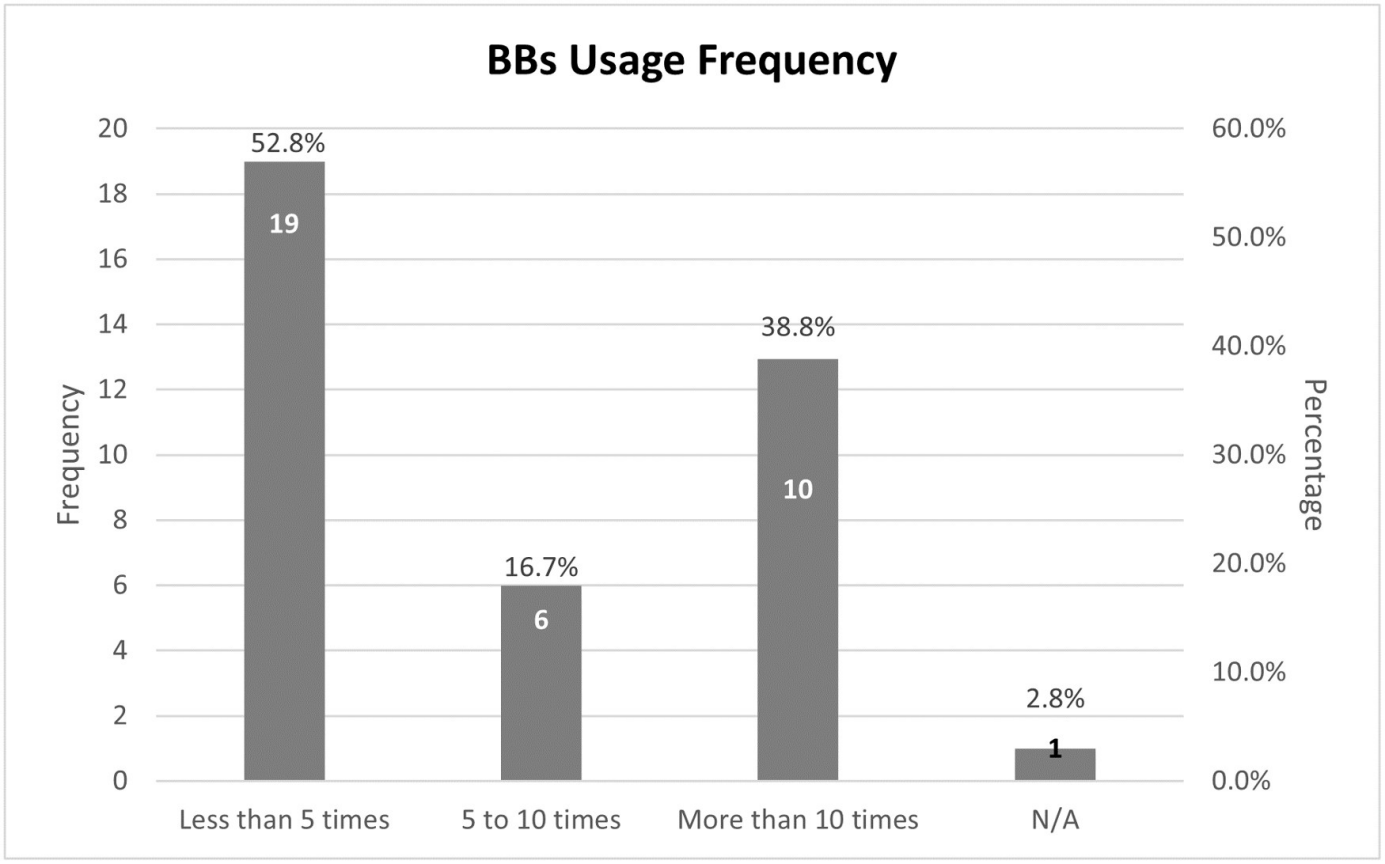
Distribution of beta-blockers usage frequency.

Thirty-six out of four hundred and sixty-one participants reported using beta-blockers (See Table 2). Among the 36 students who reported using BBs, 19 students (52.8%) reported using BBs less than 5 times, 6 students (16.7%) used BBs for 5 to 10 times, 10 students (38.8%) used BBs for more than 10 times, while one student reported not applicable to this question (Fig 1).

**Fig 2:**
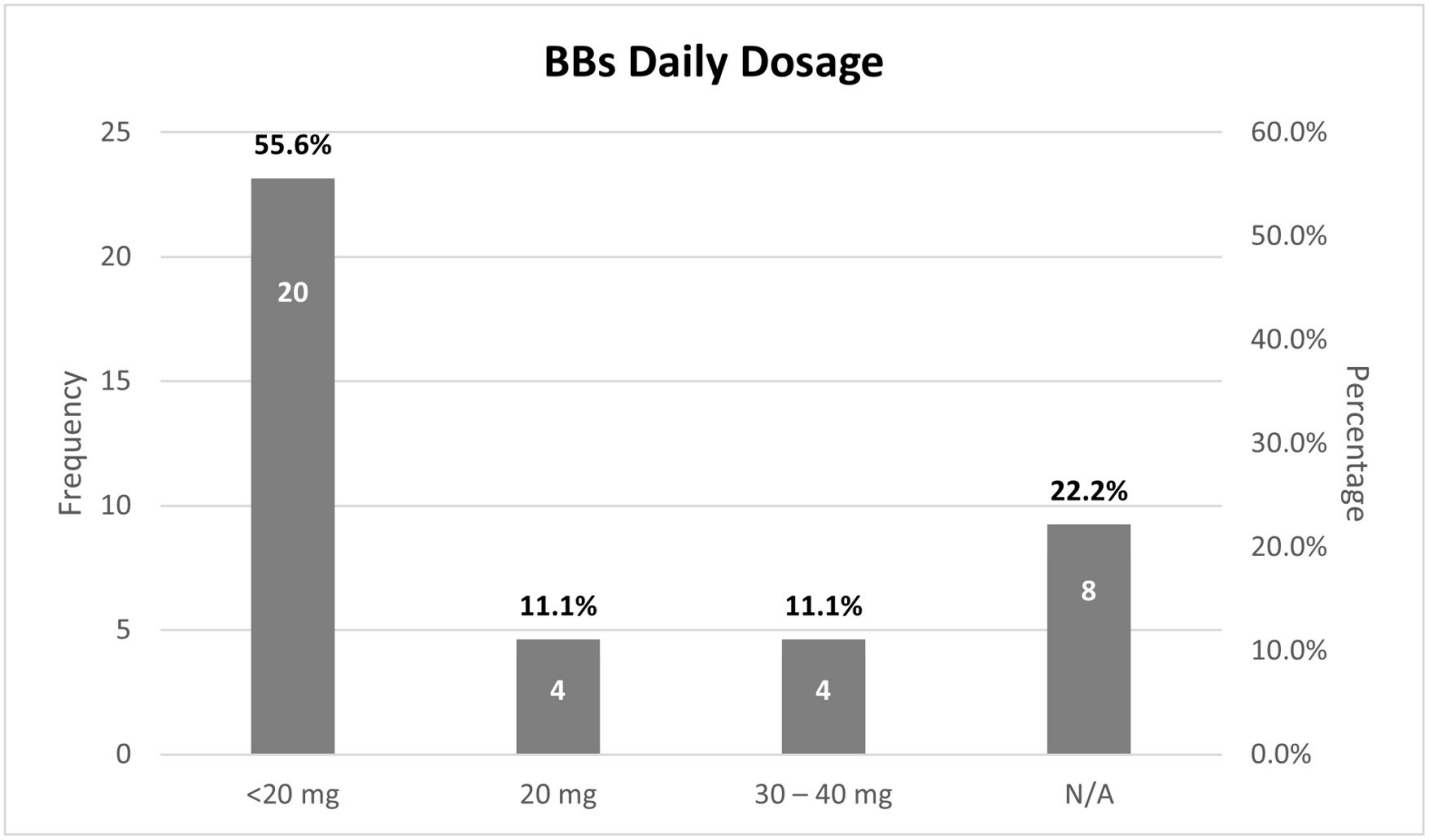
Distribution of beta-blockers daily dosage.

Regarding the BBs daily dosage among the 36 students, 20 of them (55.6%) had a less than 20 mg dose of propranolol, while 4 students (11.1%) reported the use of a 20 mg dose, and another 4 students (11.1%) reported 30 to 40 mg dose. There were 8 students (22.2%) who did not use any of the above doses and answered not applicable, meaning that they either used a higher dose or did not prefer to answer for personal reasons (Fig 2).

**Fig 3:**
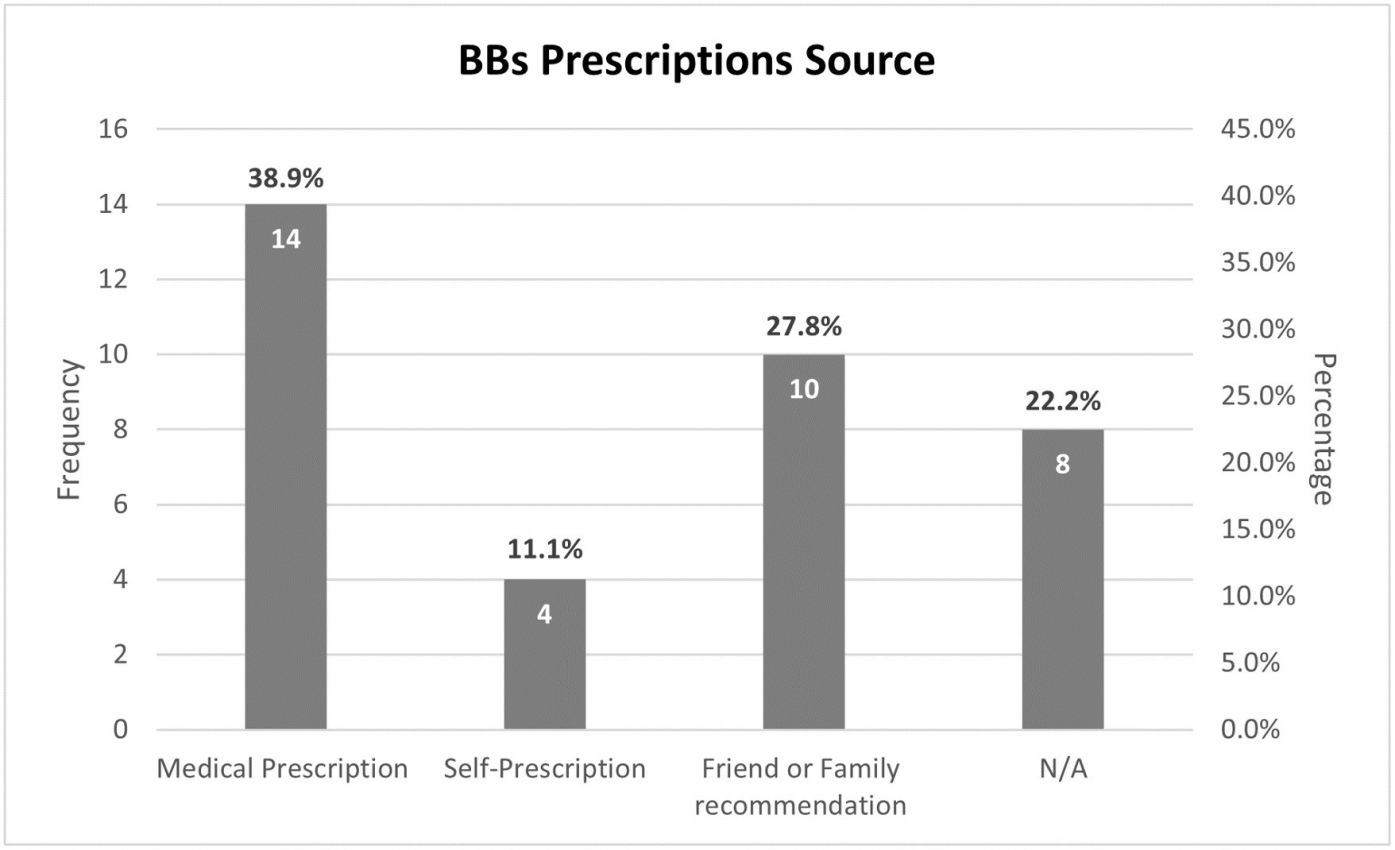
Distribution of beta-blockers prescriptions source.

Regarding the source of prescription, 14 (38.9%) of Beta-blocker users had a medical prescription, 10 (27.8%) had recommendations from friends and family, and 4 (11.1%) were self-prescriptions. It should be noted that 8 (22.2%) of users did not inform about how they accessed the drug (Fig 3).

### Reasons for using beta-blocker

The students were asked about the reasons why they use BBs. The most selected reason was "to improve performance," chosen by 16 students. "To reduce stress/anxiety" was selected by 15 students, and "To reduce social pressure and trembling" was selected by 7 students. Only 4 students reported using BBs for their blood pressure, while one did not mention the reason (See Table 3).

**Table 3:** Distribution of reasons and situation for using beta-blocker.

Using BB	Selected	Not Selected
**Reasons for Using BB**		
Improve performance	16	20
Reduce stress/anxiety	15	21
Reduce social pressure	7	29
Reduce trembling	7	29
Improve blood pressure	4	32
Other	1	35
**Situations for Using BB**		
Presentations	15	21
Oral exams	11	25
Paper exams	10	26
Social gathering	6	30
Palpitations	1	35
Heart disease	1	35
Blood pressure	1	35
Nervous agitation	1	35
Other	2	34

### Situations for using beta-blockers

Beta-blockers were most used for presentations, selected by 15 students. Oral and paper exams were selected by 11 and 10 students, respectively. Six students reported using BBs 6 times for social gatherings. Four students, each of whom had reported using beta-blockers for (heart disease, palpitations, blood pressure, or nervous agitation). Two others did not report their situations for using beta-blockers (See Table 3).

### Beta-blocker use with gender analysis

The Fisher’s Exact test showed a non-significant value for the use of BBs based on gender (P = 0.068) among 36 users of BBs. 35 of them were female, while one male used BBs (See Table 4).

**Table 4:** Factors affecting beta-blocker use.

Factors	Beta-Blocker Use, n (%)		*P*-Value
	Yes (n = 36)	No (n = 425)	
**Gender**			0.068
Male	1 (2.8)	57 (13.4)	
Female	35 (97.2)	368 (91.7)	
**Colleges**			0.666
PHHI	22 (61.1)	244 (57.4)	
Other health colleges	14 (38.9)	181 (42.6)	
**Academic Year**			0.192
1^s t^& 2^nd^ year	17 (47.2)	196 (46.1)	
3^rd^ & 4^th^ year	12 (33.3)	185 (43.5)	
5^th^, 6^th^, &7^th^ year	7 (19.4)	44 (10.4)	
**Grade Point Average**			0.985
Excellent	24 (66.7)	284 (66.8)	
Good to very good	12 (33.3)	141 (33.2)	
**Mental Condition History**			0.008
Yes	7 (19.4)	25 (5.9)	
No	(29 (80.6)	(400) (94.1)	

### Beta-blocker use with college analysis

The results of the chi-square test revealed that there was no significant association between the use of beta-blockers and a particular college (P = 0.666). A majority of the beta-blocker users (61.1%) were enrolled in the PHHI college, while the remaining users (38.9%) belonged to the colleges of Applied Medical Sciences, Nursing, Pharmacy, Medicine, and Dentistry (See Table 4).

### Beta-blocker use with academic year analysis

The chi-square test results showed that there was no significant association between the use of beta-blockers and the academic year of the students (P = 0.192). Among the beta-blocker users, the majority (47.2%) were in their first and second years, followed by 33.3% in their third and fourth years, and 19.4% in their fifth, sixth, and seventh years of study (See Table 4).

### Beta-blocker use with grade point average analysis

The Chi-Square test showed a non-significant relationship between BBs used and GPA (P = 0.985). Two-thirds (66.7%) of the BBs users with excellent GPA, while a third (33.3%) have a good and very good GPA (See Table 4).

### Beta-blocker use with history of mental conditions analysis

The Fisher’s Exact test revealed a significant association between beta-blocker use and mental conditions (P = 0.008). Of the 36 students who used beta-blockers, seven (19.4%) had a history of mental illness, while 29 (80.6%) did not (See Table 4).

### Social Phobia Inventory with gender analysis

The chi-square test showed a significant value between SPIN based on Gender (P = 0.003). Among 403 (87.4%) female students, 169 (93.4%) had mild to moderate SAD and 69 (88.5%) had severe to very severe SAD. Among 58 (12.6%) male students, 12 (6.6%) had mild to moderate SAD and 9 (11.5%) had severe to very severe SAD (See Table 5).

**Table 5:** Factors affecting Social Phobia Inventory (SPIN).

Factors	SPIN Categories, n (%)			*P*-Value
	No SPIN (n = 202)	Mild To Moderate SPIN (n = 181)	Sever To Very Sever SPIN (n = 78)	
**Gender**				0.003
Male	37 (18.3)	12 (6.6)	9 (11.5)	
Female	165 (81.7)	169 (93.4)	69 (88.5)	
**Colleges**				0.130
PHHI	120 (59.4)	109 (60.2)	37 (47.4)	
Other health colleges	82 (40.6)	72 (39.8)	41 (52.6)	
**Academic Years**				0.390
1^st^ & 2^nd^ year	87 (43.1)	89 (49.2)	37 (47.4)	
3^rd^ & 4^th^ year	87 (43.1)	78 (43.1)	14 (7.7)	
5^th^, 6^th^, &7^th^ year	37 (47.4)	32 (41)	9 (11.1)	
**Grade Point Average**				0.711
Excellent	137 (67.8)	122 (67.4)	49 (62.8)	
Good to very good	65 (32.2)	59 (32.6)	29 (37.2)	
**Mental Condition**				0.015
Yes	7 (3.5)	15 (8.3)	10 (11.5)	
No	195 (96.5)	166 (91.7)	68 (88.5)	

### Social Phobia Inventory with colleges analysis

The Chi-square test showed no significant relationship between social phobia and a specific college (P = 0.130). Out of 259 students, 146 were from PHHI College, 109 had mild to moderate SAD and 37 had severe to very severe SAD. The remaining 113 students were from applied medical sciences, nursing, pharmacy, medicine, and dentistry, 72 had mild to moderate SAD and 41 had severe to very severe SAD (See Table 5).

### Social Phobia Inventory with academic year analysis

The Pearson Chi-Square test indicated that there was no significant relationship between SPIN (Social Phobia Inventory) scores and the academic years of the students (P = 0.390). Out of the total of 259 students, 126 were enrolled in their first and second years, 110 were in their third and fourth years, and 23 were in their fifth, sixth, and seventh years. Among the first- and second-year students, 89 exhibited mild to moderate Social Anxiety Disorder (SAD) symptoms, while 37 experienced severe to very severe SAD symptoms. Similarly, among the third- and fourth-year students, 78 had mild to moderate SAD symptoms, and 32 had severe to very severe SAD symptoms. Among the students in their fifth, sixth, and seventh years, 14 had mild to moderate SAD symptoms, while 9 experienced severe to very severe SAD symptoms (See Table 5).

### Social Phobia Inventory with grade point average analysis

The Pearson Chi-Square test revealed no significant relationship between SPIN (Social Phobia Inventory) scores and GPA (Grade Point Average) (P = 0.711). Among the students who reported having Social Anxiety Disorder (SAD), nearly two-thirds (171) achieved an excellent GPA. Within this group, 122 students had mild to moderate SAD, while 29 students experienced severe to very severe SAD. Additionally, among the students with a good and very good GPA, 88 individuals had mild to moderate SAD, and 29 students reported severe to very severe SAD (See Table 5).

### Social Phobia Inventory with mental condition history analysis

The analysis of chi-square indicates a significant effect between a history of mental conditions and the presence of social anxiety disorder (P = 0.015). Among 461 students, 32 (6.9%) students reported a history of mental conditions, while 429 (93.1%) did not report mental condition history. Among those who reported mental condition history, 15 (8.3%) had mild to moderate SAD, 10 (11.5%) had severe to very severe SAD, and 7 (3.5%) had no social anxiety (See Table 5).

## Discussion

The study’s findings showed that there was no significant correlation between social anxiety disorder (SAD) scores and the prevalence of beta-blockers (BBs) use due to the ineffectiveness of beta-blockers in treating psychic symptoms associated with the central nervous system (CNS), such as anxiety, fear, and avoidance [15].

This study found that almost two-thirds of students (56.2%) have reported some degree of Social Phobia. Mild to moderate levels of SAD was reported by 39.3%, while 16.9% exhibited severe to very severe symptoms. Although there have been global studies on the prevalence of SAD among undergraduate medical students, comparing the results was difficult due to variations in research methods, participant demographics, cultural factors, and societal influences. Our findings were similar to those of a study involving 504 medical students from Taibah University, Saudi Arabia, using the SPIN questionnaire, revealing that 13.5% of participants experienced severe to very severe SAD [16]. Findings from the present study were higher than other studies conducted in India, Saudi Arabia, and Iran, where the prevalence of SAD was estimated at 7.8%, 16.3%, and 7.9%, respectively [17–19]. The sample size and differences in the statistical populations, cultural backgrounds of the participants, and variations in the periods when the studies were conducted might have led to different results.

Upon examining the relationship between varying degrees of SAD and other variables in this study, we observed a significant connection between SAD presence and gender differences. 87.4% of females experienced very severe social phobia, while 12.6% had mild symptoms compared to males. This observation aligns with the DSM-5 statement, which indicates that SAD prevalence is higher among females, especially during adolescence [1]. In contrast to the previous finding, many studies conducted in Iran, Canada, Malaysia, and even Saudi Arabia did not report significant gender-based differences in SAD scores [2, 20–22]. However, this difference may be attributed to multiple factors contributing to results variations. For instance, using different assessment tools to evaluate the prevalence of SAD could influence the outcome and the diverse socio-demographic and cultural norms across these communities.

In this study, we found no effect of different GPAs on SAD severity (P = 0.711), while earlier Saudi research suggested that higher SAD scores were associated with lower academic achievement among medical students [7, 16, 23]. The variations observed across the studies might result from a range of factors, including the demographic characteristics of the participants. Notably, the majority of the current study participants with high GPA in this study were from the PHHI college, which is known to employ a more theoretical approach to learning and assessment, such as written exams, in opposition to practical approaches that often use Objective structured clinical examination (OSCE)/Objective structured practical examination (OSPE) and other oral exams. Therefore, situations that demand students to confront SAD, specifically performance anxiety and interact with the public, may be relatively less frequent in PHHI compared to other colleges, such as medical and dental, that prioritize applied education.

Additionally, the study found a significant association between SAD and the presence of other mental conditions (P = 0.015). In a study conducted by Bassiony in Saudi Arabia, 58% of patients with SAD had another concurrent psychiatric disorder, with 41% experiencing depression and 92.5% developing it after SAD onset [24]. Among those with severe SAD, 69% had depression, compared to 35% of patients with mild or moderate SAD [24]. SAD increases the likelihood of experiencing depressive symptoms, and the fear subtype of social phobia is strongly linked to depression [25]. Several clinical and nonclinical studies have reported that SAD predicts a heightened risk for depression [4, 26–29].

The prevalence of BBs users in the sample studied at UQU of this study was found to be 7.8%, which is lower when compared to 14.4% of users in a study conducted at King Saud bin Abdulaziz University for Health Sciences [14]. This difference in prevalence may be attributed to varying factors such as prescribing practices between the two studies. Furthermore, the current study found 11.1% of individuals were self-prescribed BBs. This rate is relatively lower than the rate reported in a previous study by Albaraa Alsini, 2021 [30]. Regarding the doses of BBs, the current study found that the highest prevalence was for doses less than 20 mg (55.6%). This contrasts with another study which found that the most prevalent dose was 20 mg (9.5%). To minimize somatic side effects, it is recommended to administer BBs within the safe dosage range of 10 to 20 mg [8].

In addition to using BBs the study results revealed that the causative stimulants to use propranolol among health students were to improve performance (44.4%) and reduce stress and anxiety (41.7%). This finding differs from another study, which reported that 70% of medical students used propranolol to alleviate educational stress and anxiety [31]. These results suggest that the reasons for using propranolol may vary depending on the academic discipline. Considering the study’s findings, a significant proportion of health students use BBs, particularly propranolol, before presentations to manage their performance anxiety. Specifically, 41.7% of the students used propranolol before a presentation, consistent with previous research that reported BBs as a commonly used coping mechanism for individuals experiencing performance anxiety. Interestingly, the rate of propranolol usage before presentations was lower than that before oral exams (ODCE), which was reported to be 70.6% in another study [14]. These results suggest that the type of academic challenge students face may influence their decision to use BBs. The findings of Butt et al’s study in 2016 raise concerns about the potential long-term effects of beta-blocker usage before exams. Specifically, they found that students who took beta-blockers before exams had a higher risk of taking antidepressants in the future and were more likely to attempt suicide than non-users [32]. Based on this, it is crucial to raise awareness that beta-blocker users before exams may be more vulnerable to psychological issues and require special attention [30]. In line with this concern, the current study found that the prevalence of beta-blocker use for mental conditions was significantly higher among the target group (19.4%) than the rate among medical students at the University of Jeddah (1.2%) [14]. This raises questions about the potential link between beta-blocker use and mental health disorders which suggests that there may be a higher incidence of mental health disorders among the study participants or a greater awareness of the benefits of beta-blockers in managing mental health conditions. Further research is needed to understand the underlying factors contributing to this disparity in beta-blocker use between the two groups, particularly in the context of mental health treatment in Saudi Arabia. By doing so, healthcare professionals can develop appropriate interventions to address mental health disorders and reduce the reliance on beta-blockers as a coping mechanism. Medical schools could benefit from offering support programs for their students to manage their mental health conditions and reduce their reliance on unprescribed medications such as BBs.

The present study sheds light on the prevalence of self-reported propranolol use among medical students, which is a topic that has not been extensively researched until recently. This study was the first in Saudi Arabia to investigate the use of beta-blockers with the prevalence of social anxiety. Moreover, demographic information was identified as potential confounding variables and was included as covariates in statistical analyses. This approach allowed the researchers to control for potential demographic differences that may have influenced the relationship between beta-blocker use and social anxiety. However, there are some limitations, one is that the study relies on self-reported data, which may be subject to recall bias or social desirability bias that might have led to underreporting of the results [33]. Or that the convenience sampling method was not the most suitable method to detect the true prevalence of using beta-blockers due to insufficient time and resource availability. It also does not examine beta-blockers effectiveness in treating SAD, which limits understanding the potential benefits or drawbacks of beta-blocker usage for managing SAD symptoms.

## Conclusions

In this study conducted at UQU Makkah, it was observed that the utilization of beta-blockers (BBs) among health students was relatively low, whereas the prevalence of Social Anxiety Disorder (SAD) was found to be higher than other students from previous studies. Although no significant relationship was identified between the two variables, the elevated prevalence of SAD highlights the need for a comprehensive student support program aimed at offering guidance and care to students affected by SAD, as well as addressing the underlying causes of this disorder. The suggested program should encompass counseling services, workshops, and additional resources that empower students to manage their mental health and overall well-being. By prioritizing student support initiatives, Umm Al-Qura University can foster a more nurturing and inclusive learning environment that fosters academic achievement and personal development.

## Supporting information

S1 Data (XLSX)
